# Inhibitory effects of lapachol on rat C6 glioma in vitro and in vivo by targeting DNA topoisomerase I and topoisomerase II

**DOI:** 10.1186/s13046-016-0455-3

**Published:** 2016-11-16

**Authors:** Huanli Xu, Qunying Chen, Hong Wang, Pingxiang Xu, Ru Yuan, Xiaorong Li, Lu Bai, Ming Xue

**Affiliations:** Department of Pharmacology, School of Basic Medical Sciences, Capital Medical University, No.10 Youanmenwaixitoutiao, Fengtai District Beijing, 100069 China

**Keywords:** Lapachol, C6 glioma, Topoisomerase I, Topoisomerase II

## Abstract

**Background:**

Lapachol is a natural naphthoquinone compound that possesses extensive biological activities. The aim of this study is to investigate the inhibitory effects of lapachol on rat C6 glioma both in vitro and in vivo, as well as the potential mechanisms.

**Methods:**

The antitumor effect of lapachol was firstly evaluated in the C6 glioma model in Wistar rats. The effects of lapachol on C6 cell proliferation, apoptosis and DNA damage were detected by 3-(4,5-dimethylthiazol-2-yl)-5-(3-carboxymethoxyphenyl)-2-(4-sulfophenyl)-2H-tetrazolium (MTS)/ phenazinemethosulfate (PMS) assay, hoechst 33358 staining, annexin V-FITC/PI staining, and comet assay. Effects of lapachol on topoisomerase I (TOP I) and topoisomerase II (TOP II) activities were detected by TOP I and TOP II mediated supercoiled pBR322 DNA relaxation assays and molecular docking. TOP I and TOP II expression levels in C6 cells were also determined.

**Results:**

High dose lapachol showed significant inhibitory effect on the C6 glioma in Wistar rats (*P* < 0.05). It was showed that lapachol could inhibit proliferation, induce apoptosis and DNA damage of C6 cells in dose dependent manners. Lapachol could inhibit the activities of both TOP I and II. Lapachol-TOP I showed relatively stronger interaction than that of lapachol-TOP II in molecular docking study. Also, lapachol could inhibit TOP II expression levels, but not TOP I expression levels.

**Conclusion:**

These results showed that lapachol could significantly inhibit C6 glioma both in vivo and in vitro, which might be related with inhibiting TOP I and TOP II activities, as well as TOP II expression.

## Background

Glioma is the most common primary brain tumor and accounts for about 40% of all primary brain tumors. Despite advances in malignant glioma treatment in recent years, the prognosis of patients with malignant glioma is extremely poor [1]. Temozolomide (TMZ), an oral DNA-alkylating agent that can cross the blood-brain barrier, is the major chemotherapeutic drug used in clinical for the treatment of malignant gliomas [2]. However, malignant glioma cells quickly develop TMZ resistance and the long-term clinical benefits of TMZ are poor [3]. Thus, developing new drugs that can improve therapeutic benefit and prolong survival of malignant glioma patients is urgently needed.

Naphthoquinone is an important class of naturally occurring active ingredients with unique physical and chemical properties and pharmacological effects [4]. They are widely used as anticarcinogenic, antibacterial, antimalarial, and fungicidal agents [5]. Some well-known anticancer drugs (*e.g.* doxorubicin, mitomycin and mitoxantrone) possess a quinonoid structure. Lapachol (4-hydroxy-3-(3-methylbut -2-enyl) naphthalene-1,2-dione) is a naphthoquinone that can be isolated from many species of *Bignoniaceae* family [6]. Lapachol has a long history of anticancer activity that stretches back to the 1970’s [7]. The cytotoxicity of lapachol and its derivatives were evaluated in many tumor cells, such as oesophageal cancer cells [8], Ehrlich’s carcinoma [9], K562 leukemic cells [9], A549 non-small cell lung cancer, PC-3 prostate cancer, SKMEL-28 melanoma, LoVo colon cancer cell lines, and human glioma lines (U373 and Hs683) [10], as well as in several mouse models [11]. It was reported that lapachol did not show any carcinogenic activity [12]. Studies on the action mechanism of naphthoquinone and its derivatives have shown their inhibitory effects on DNA topoisomerases [13–15].

It was reported that some 1,4-naphthoquinone derivatives and lapachol showed strong cytotoxicity on glioma cells [10, 16]. We have previously studied the in vivo lapachol metabolism using a sensitive LC-ESI–MS^n^ method [17], and found that lapachol was able to cross the blood brain barrier, indicating that it might be effective in treating malignant glioma. Since the effect of lapachol on malignant glioma has not been extensively studied, we evaluated the effect of lapachol on malignant glioma and the potential mechanisms in this study.

## Methods

### Chemicals and reagents

Lapachol [2-hydroxy-3-(3 -methyl-2 -butenyl-)-1,4- naphthoquinone] was purchased from Sigma Aldrich (St. Louis, MO, USA). DMEM medium and fetal bovine serum were purchased from Invitrogen Co (Carlsbad, CA). 3-(4,5-dimethylthiazol-2-yl)-5-(3-carboxymethoxyphenyl)-2-(4-sulfophenyl)-2H -tetrazolium (MTS)/phenazinemethosulfate (PMS) assay kit and Annexin V-FITC/PI staining kit were purchased from Promega (Madison, WI, USA). Comet assay kit was bought from Nanjing KeyGEN BioTECH. Co. Ltd. in China. Topoisomerase I and II drug screening kits were bought from Topogen Inc. (USA). Enzyme-linked Immunosorbent Assay Kits for Topoisomerase I (TOP I) and TOP II were bought from Cloud-clone Corp. (Houston, USA). All other chemicals used were of the highest purity available from commercial sources.

### Cells and cell culture conditions

The C6 glioma cell line was obtained from the Cell Resource Center of Peking Union Medical College (Beijing, China). The cells were cultured in DMEM supplemented with 10% fatal bovine serum at 37 °C in a humidified incubator containing 5% CO_2_. Cell viability was evaluated with Typan blue staining. Only cell suspensions with more than 95% viability were acceptable for implantation.

### Establishment of rat C6 glioma model and lapachol treatment

Male Wistar rats weighing 160–180 g were used. After anesthesia with 10% chloral hydrate (3 mL/kg), the head of the rat was fixed in a stereotactic apparatus and the surgery area was disinfected with iodine. Then, the bregma was exposed by making a midline incision on the dorsal aspect of the head. Then, a 1 mm diameter hole was drilled in the cranial bone 1 mm posterior to the bregma and 3 mm lateral to the sagittal suture. About 3 × 10^6^ C6 cells in 15 μL phosphate-buffered saline (PBS) were injected using a 25 μL microsyringe in 10 min. The tip of the microsyringe was inserted 6 mm beneath the dura, and then withdrawn 1 mm. After injection, the syringe remained in the brain for an additional 5 min, and then retracted slowly. The holes were sealed with bone wax and the wounds were closed. The rats were allowed to recover for 7 days under standard conditions (12-h light/dark, 22 ± 2 °C) with food and water adlibitum.

Seven, 14 and 20 days after implantation, tumors in the brains of the rats were detected by Bruker 7.0 T Micro-MRI using a T2W RARE sequence with parameters as follows: TR/TE 3000/15 ms, slice thickness 1.0 mm, slice gap 1.0 mm, FOV 33 × 33 mm, Matrix 256 × 256, flip angle 180°, time 4.8 min. The maximum anteroposterior diameter (L), width (W) and height (H) in the largest enhanced areas on the horizontal and coronal planes were measured. The tumor volumes (*V*) were calculated as follows:

$$ \mathrm{V}=\left(\frac{4}{3}\times \uppi \times \mathrm{L}\times \mathrm{W}\times \mathrm{H}\right)\times \frac{1}{8}\left({\mathrm{mm}}^3\right) $$

Then, rats were sacrificed by decapitation and the brains tissues were isolated. The tissues were formalin-fixed, paraffin-embedded and then cut into 10 μm-thick sections. The sections were subjected to hematoxylin/eosin (HE) staining and immunochemical staining for glial fibrillary acidic protein (GFAP) as previously described [18]. The sections from each animal were analyzed by a pathologist.

Seven days after glioma implantation, the rats were randomly divided into five groups: control group (0.5% CMC-Na, *n* = 8), TMZ group (25 mg/kg, *n* = 10), and different lapachol groups (low dose, 5 mg/kg; middle dose, 25 mg/kg; high dose, 100 mg/kg, *n* = 8 in each group). The drugs were given by intragastric administration once daily. The body weight was recorded each day. Seven and 13 days after treatment, tumors in the brains of the rats were detected by Bruker 7.0 T Micro-MRI using a T2W RARE sequence with parameters mentioned above. Tumor volumes were calculated as mentioned above. All experiments were performed with the approval from the Capital Medical University Ethics Committee in Beijing, China (number 37363).

#### Anti-proliferation assay

We investigated the effects of lapachol against C6 cells by MTS/PMS assay. Cells in logarithmic growth phase were plated in 96-well plates at a density of 3000 cells/well for 24 h. The cells were incubated with 1.25, 2.5, 5, 10, 20 μM of lapachol for 48 h. Then, MTS and PMS mixed at the ratio of 20:1 were immediately added to the culture medium. After 2 h, formazan production was analyzed at 490 nm by a Thermo Scientific™ Multiskan™ GO Microplate Spectrophotometer. The inhibitory rates and half inhibitory concentration (IC_50_) was then calculated.

#### Apoptotic analysis

Hoechst 33258 staining was used for detecting the apoptotic morphology of the treated cells. Cells were seeded in 96-well plates at 3000 cells/dish overnight and then treated with 1, 5, 10 μM of lapachol for 48 h. Then the cells were washed and stained with 10 μg/mL of Hoechst 33358 for 20 min at 37 °C. After washing with PBS, morphologic changes of the cells were observed under a fluorescence microscope and photographed.

Annexin V-FITC/PI staining was used for detecting apoptosis rates. Briefly, cells were seeded in 60 mm culture dishes at 3 × 10^5^ cells/mL and incubated overnight. The cells were treated with 1, 5, 10 μM of lapachol for 48 h. Then cells were collected and resuspended in 500 μL detection buffer, followed by adding 5 μL PI and 5 μL Annexin V-FITC to the detection buffer. Then cells were incubated for 15 min in the dark and analyzed by a BD FACS Calibur™ system.

#### Comet assay

Comet assay was performed using the Comet Assay kit (KEYGEN BIOTECH. CO., Nanjing, China) according to the manufacture’s instruction. After treatment with 1, 5, 10 μM of lapachol for 48 h, the cells were harvested and resuspended in 1 mL ice-cold PBS. Then 10 μL cell suspension (10^4^ cells) were mixed with 75 μL low-melting agarose at 37 °C for 20 min, and then added to clean microscope slides, which had been covered with 100 μL 0.75% normal-melting agarose, and the gels were solidified at 4 °C for 10 min. Then the slides were lysised for 1-2 h, immerged in alkaline buffer (1 mM EDTA and 300 mM NaOH), and then subjected to electrophoresis at 25 V for 30 min. Finally, 2 μL PI was dropped onto each slide and covered with a clean cover slip and then observed by a fluorescent microscope. A total of 50 C6 cells were randomly analyzed with an image analysis system (Komet 5.5, Kinetic Imaging Ltd., UK) and DNA migration was determined by measuring the “tail intensity” (% tail DNA).

#### TOP I and II mediated DNA relaxation assays

The effects of lapachol on TOP I and TOP II activities were assessed by the conversion of supercoiled pBR322 DNA to its relaxed form using topoisomerase I and II drug screening kits (Topogen). The TOP I activity was studied in a 20 μL reaction system including 1 μL human TOP I, variable volume of lapachol (finaly concentration: 1, 10, 100 μM) or camptothecin (50 μM), 4 μL 5 × complete assay buffer, 1 μL pBR322 DNA, and variable volume H_2_O (to make up to volume). The reaction mixtures were incubated at 37 °C for 30 min. The reactions were stopped with 2 μL 10% SDS and then incubated with proteinase K (50 μg/mL) at 37 °C for 15 min. Then the samples were run on a 1% agarose gel with 0.5 μg/mL ethidium bromide at 50 V. The gel was destained in water before being photographed under UV-light. The known camptothecin was used as the positive control. Inhibition of TOP II activity by lapachol was performed using a similar procedure according to the manufacture’s instruction. The known TOP II poisons etoposide was used as the positive control.

#### Molecular docking studies

Molecular docking study was performed for comparing the mode of action of lapachol-TOP I and lapachol-TOP II using MOE 2010 software package. Crystal structures of TOP I-ligand complex (PDB entry: 1SC7, 3.0 Å) and TOP II-ligand complex (PDB entry: 1ZXM, 1.87 Å) were used. All water molecule ligands were removed and the docking active pockets were defined by the ligand molecules. The detail docking parameters of TOP I were as follows: placement method (Triangle Matcher), the first scoring function rescoring (Affinity dG), and the saved poses (100); the refinement (forcefield), the second refinement scoring function rescoring (London dG) and the saved poses (30). The detail docking parameters of TOP II were as follows: placement method (Triangle Matcher), the first scoring function rescoring (London dG), and saved poses (30); the refinement (forcefield), the second refinement scoring function rescoring (none), the refinement saved poses (10). To verify whether MOE software was suitable for docking TOP I and TOP II, the ligand conformations of 1ZXM and 1SC7 were withdrawn and re-docked to the active pockets. The first 10 and 30 conformations of the docking scores were saved and the root-mean-square deviation (RMSD) values of docking conformation and initial conformation were calculated. Then, the ligand molecules in the crystal structures were re-docked to the defined active pockets, and the scores of ligand conformation after docking and original conformation in the crystal structures were calculated.

#### Detection of TOP I and TOP II levels in C6 cells

TOP I and TOP II expression levels in the cells were determined by Enzyme-linked Immunosorbent Assay Kits for TOP I and TOP II (Cloud-clone Corp.). Briefly, after treatment with 1, 5, 10 μM of lapachol for 48 h, the cells were collected and subjected to ultrasonication for 3 times. The supernatants were collected and protein concentrations were determined. Then, 100 μL each of dilutions of standard (20 ng/mL, 10 ng/mL, 5 ng/mL, 2.5 ng/mL, 1.25 ng/mL, 0.625 ng/mL, 0.312 ng/mL), blank and samples were added into the plate wells and incubated for 2 h at 37 °C. Then 100 μL Detection Reagent A working solution was added to each well. The wells were incubated for 1 h at 37 °C. After washing, 100 μL of Detection Reagent B working solution was added to each well. The wells were incubated for 30 min at 37 °C. After washing, 90 μL Substrate Solution was added and the wells were incubated for 20 min at 37 °C. Finally, 50 μL Stop Solution was added and the optical density (OD) was detected at 450 nm. A standard curve was then constructed by plotting the mean OD and concentration for each standard. The concentrations of TOP I and TOP II in the samples were then calculated by comparing the OD of the samples to the standard curve.

#### Real-time polymerase chain reaction (RT-PCR) for TOP I and TOP II

After incubation with 1, 5, 10 μM of lapachol for 48 h, total RNA of the cells was extracted using TRIzol (Invitrogen, Carlsbad, CA, USA) according to the manufacturer’s instructions. Reverse transcription was carried out using the PrimeScript™ RT Master Mix (TaKaRa, Japan). Real-time PCR was conducted using the SYBR Green dye (TaKaRa, Japan). The primers were as the following: TOP I, 5′-CTCAGCCGTTTCTGGAGTCT-3′ (forward) and 5′-TCAGCATCATCCTCAT CTCG-3′ (reverse); TOP IIα, 5′-ACAATTGGCCGCTAAACTTG-3′ (forward) and 5′-GCGAGTGTGCTGGTCACTAA-3′ (reverse); GAPDH, 5′-TCACCAGGGCTG CTTTTAAC-3′ (forward) and 5′-GACAAGCTTCCCGTTCTCAG-3′(reverse); Real-time PCR was performed in triplicate on CFX96 Real Time PCR Detection System (Bio-Rad, USA), and the 2^-△△T^ method was used to determine the relative gene expression.

## Results

### Lapachol could decrease tumor volumes without affecting the body weights of glioma-bearing rats

**Fig. 1:**
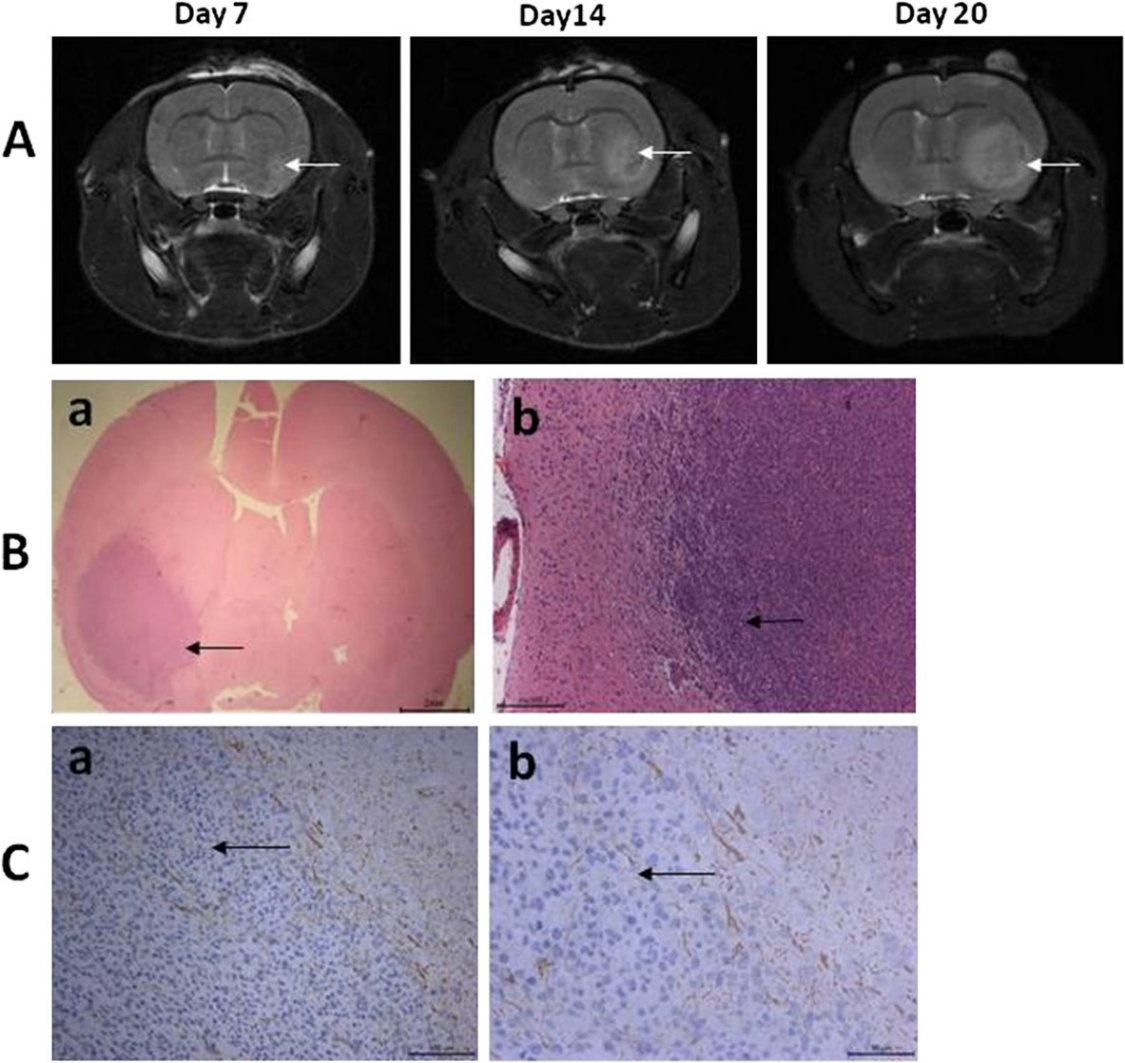
MRI, HE and immunohistochemical staining results for confirming the establishment of rat C6 glioma model. **a** MRI image of glioma-bearing rats on day 7, 14 and 20 after implantation. Glioma tumor is indicated with an *arrowhead*. **b** Representative HE staining result for the implanted gliomas. Original magnification: *a*. ×20; *b*. ×100. **c**. Representative immunohistochemical staining for GFAP. Positive cells in glioma tumor are indicated with an *arrowhead*. Original magnification: *a*. ×200; *b*. × 400

MRI, HE and immunohistochemical staining were used to confirm the successful establishment of the rat C6 glioma model. Tumors in the brains of the rats were detected by Bruker 7.0 T Micro-MRI using a T2W RARE sequence. As shown in Fig. 1a, the tumor volumes significantly increased on day 14 and day 20, compared with that on day 7. Rats with intracerebral tumor maximum diameter of >5.0 ± 0.5 mm were considered as glioma-bearing animals. HE staining showed that obvious pathologic changes were found in tumor tissue areas (Fig. 1b, arrow). GFAP is recognized as a marker for diagnosing astrocytic origin tumors. Immunohistochemical staining result showed that positive GFAP staining was observed in the tumor area, confirming that the tumor tissue was astrocytic origin (Fig. 1c, arrow).

**Fig. 2:**
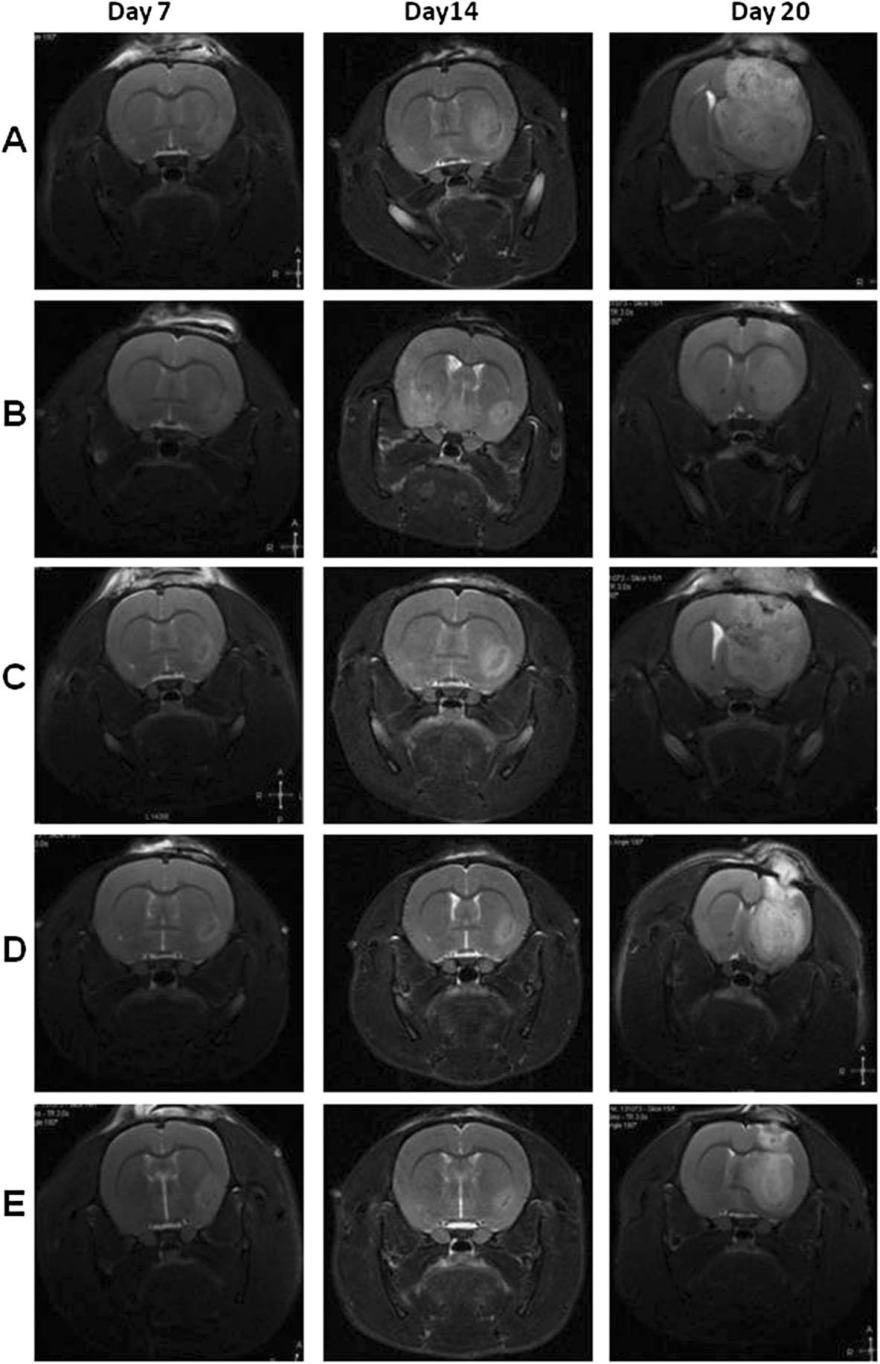
MRI examination for C6 glioma in the brains of the Wistar rats 7, 14 and 20 days after implantation. On day 7, the rats were treated with 0.5% CMC-Na solution (**a**, control), 25 mg/kg TMZ (**b**), 5 mg/kg lapachol (**c**), 25 mg/kg lapachol (**d**), and 100 mg/kg lapachol (**e**)

**Fig. 3:**
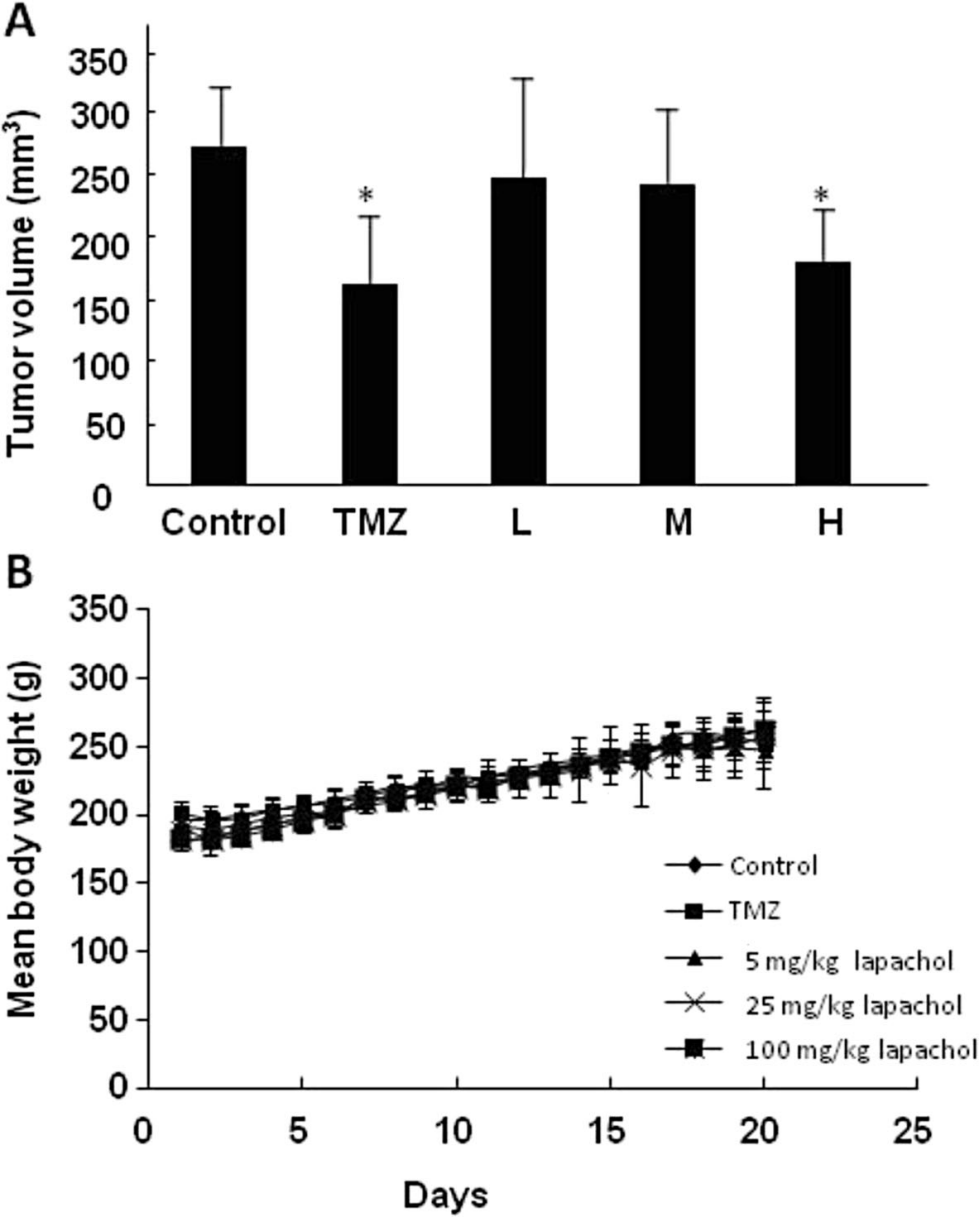
Tumor volumes (**a**) and body weight changes (**b**) of Wistar rats after being treated with TMZ and different concentrations of lapachol for 13 days. * *P* < 0.05, compared to the control group. Control: 0.5% CMC-Na solution; TMZ, 25 mg/kg; L: 5 mg/kg lapachol; M: 25 mg/kg lapachol; H: 100 mg/kg lapachol. The results are expressed as mean values ± SD

Tumors in the brains of the rats in each group were detected by MRI on day 7, 14 and 20 after implantation (Fig. 2), and the tumor volumes were calculated as mentioned above. As shown in Fig. 3a, tumor volumes in the high dose lapachol and TMZ groups were significantly decreased compared to the control group (*P* < 0.05). However, no obvious growth inhibitory effects were observed in the low dose lapachol and middle dose lapachol groups (*P* > 0.05). Moreover, compared to the control group, no obvious body weight changes were found in lapachol and TMZ treated groups (*P* > 0.05). These results suggested that high dose lapachol and TMZ treatment could significantly decrease tumor growth without affecting the body weights of the glioma-bearing rats.

### Lapachol could inhibit the proliferation of C6 cells

**Fig. 4:**
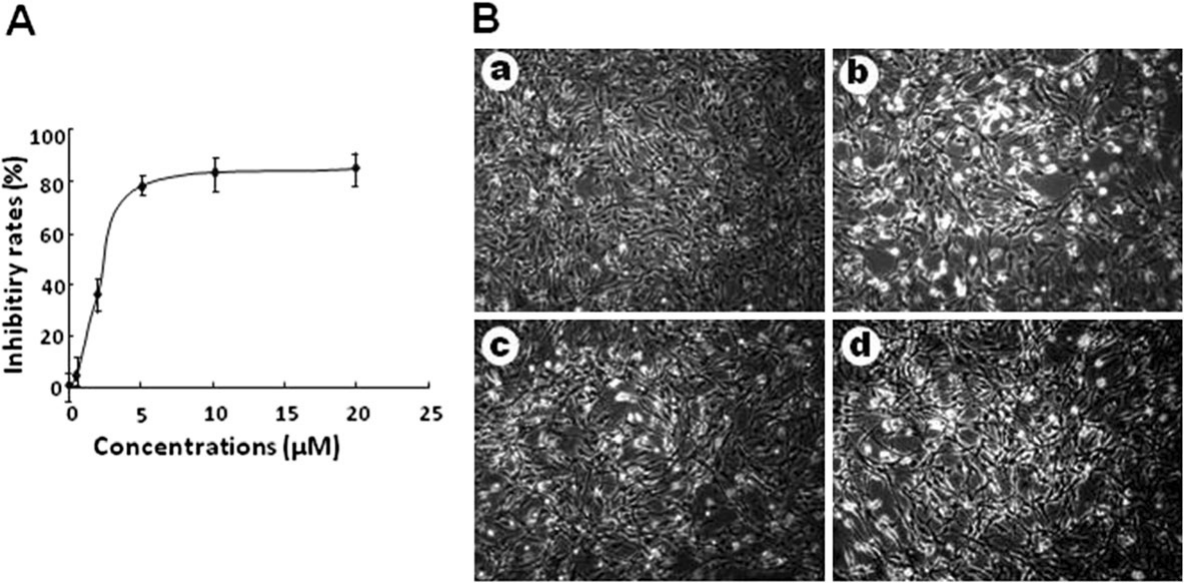
Effects of different concentrations of lapachol on proliferation and morphology of C6 glioma cells. **a** Inhibitory rates of lapachol on C6 cell proliferations. **b** Cell morphology of C6 cells after being treated with control (*a*), 1 μM lapachol (*b*), 5 μM lapachol (*c*), and 10 μM lapachol (*d*)

As shown in Fig. 4a, lapachol exerted obvious anti-proliferation effects on cancer cells in a dose-dependent manner. The IC_50_ of lapachol on C6 cells were 3.7 ± 1.4 μM. The morphology changes of C6 cells after treatment by 1.0, 5.0, and 10.0 μM of lapachol were shown in Fig. 4b. Shrinking size and elongated shape, and decreased cell number were found in the groups treated with 1.0, 5.0, and 10.0 μM of lapachol.

### Lapachol could induce apoptosis and DNA damage of C6 cells

**Fig. 5:**
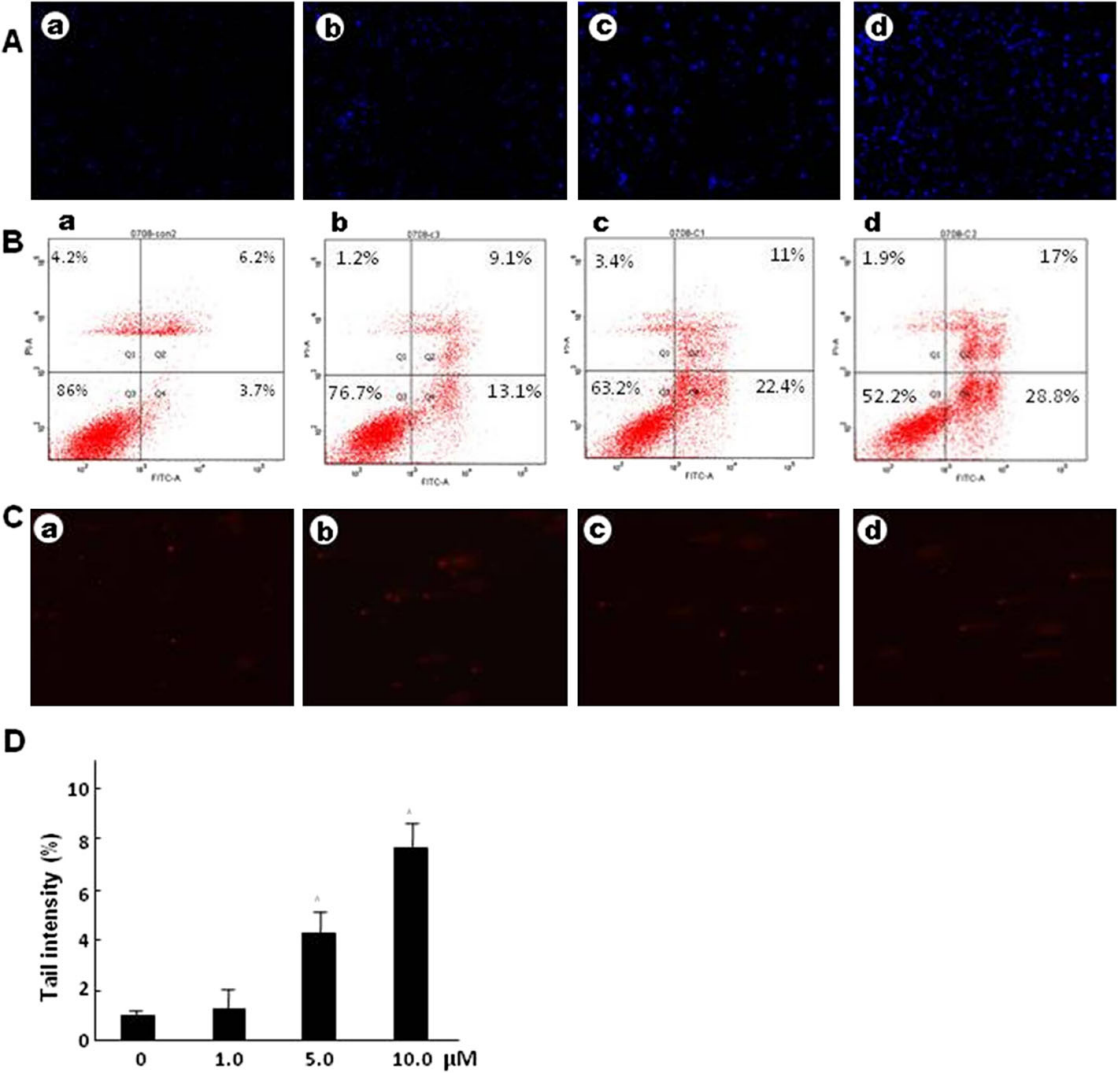
Effects of different concentrations of lapachol on apoptosis and DNA damage of C6 cells. **a** Hoechst 33258 staining for C6 glioma cells after being treated with control (*a*), 1 μM lapachol (*b*), 5 μM lapachol (*c*), and 10 μM lapachol (*d*). riginal magnification: ×200. **b** Annexin V-FITC/PI staining for apoptosis rates of cells after being treated with control (*a*), 1 μM lapachol (*b*), 5 μM lapachol (*c*), and 10 μM lapachol (*d*). **c** Comet assay result of cells after being treated with control (*a*), 1 μM lapachol (*b*), 5 μM lapachol (*c*), and 10 μM lapachol (*d*). Original magnification: ×200. **d** Tail intensity of C6 cells in the comet assay. Mean ± SD of three independent experiments. ^*^ *P* < 0.05 compared with the control

Hoechst 33258 staining result was shown in Fig. 5a. Cells in lapachol treated groups demonstrated nucleolus or cytoplasm condensation dose dependently, indicating an early apoptotic event, while cells in the control group displayed homogeneously distributed chromatin within the nucleolus.

As shown in Fig. 5b, lapachol treatment significantly increased the number of apoptotic cells (*P* < 0.05). The apoptotic rates of C6 cells were 22.2 ± 2.4%, 33.4 ± 3.7%, and 45.8 ± 9.1%, respectively after 1.0, 5.0, and 10.0 μM of lapachol treatment. Moreover, the effects of lapachol treatment on the early apoptosis were more obvious than that on the late apoptosis. These results indicated that lapachol treatment could significantly induce the apoptosis of C6 cells dose dependently.

We measured DNA damage by observing comet tails using comet assay after treatment of C6 cells with different concentrations of lapachol. As shown in Fig. 5c, obvious DNA tails can be found in the C6 cells treated with 1.0, 5.0, and 10.0 μM of lapachol. Tail intensity of DNA (% tail DNA) showed that 1.0, 5.0, and 10.0 μM of lapachol could significantly induce DNA migration in C6 cells, indicating that lapachol could induce DNA damage in a concentration-related manner (Fig. 5d).

### Lapachol could inhibit the activities of TOP I and TOP II

**Fig. 6:**
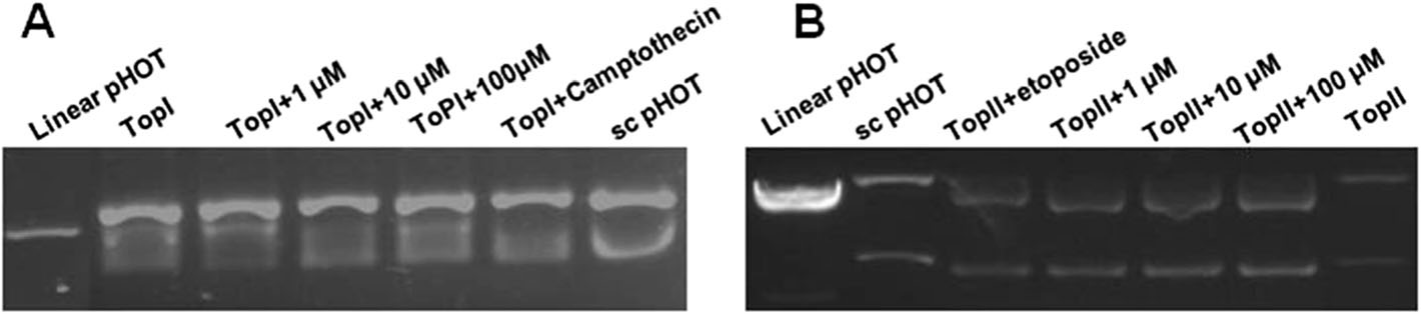
Effects of lapachol on TOP I and TOP II activities by the conversion of supercoiled pBR322 DNA to its relaxed form. TOP I and TOP II drug screening kits were used. **a** TOP I inhibition assay result by lapachol; **b** TOP II inhibition assay result by lapachol. The known TOP I inhibitor camptothecin and TOP II poisons etoposide were used as positive controls

The effects of lapachol on TOP I and TOP II activities were assessed by the conversion of supercoiled pBR322 DNA to its relaxed form. As shown in Fig. 6a, 10.0, and 100.0 μM lapachol showed obvious inhibitory effects on Top I activity, with the increased amount of supercoiled pBR322 DNA compared with the control. Fig. 6b showed that linearized pHOT DNA products were formed in the etoposide, 1.0, 10.0, and 100.0 μM lapachol treated groups, indicating that both lapachol and etoposide were Top II poisons.

### Molecular docking study

**Fig. 7:**
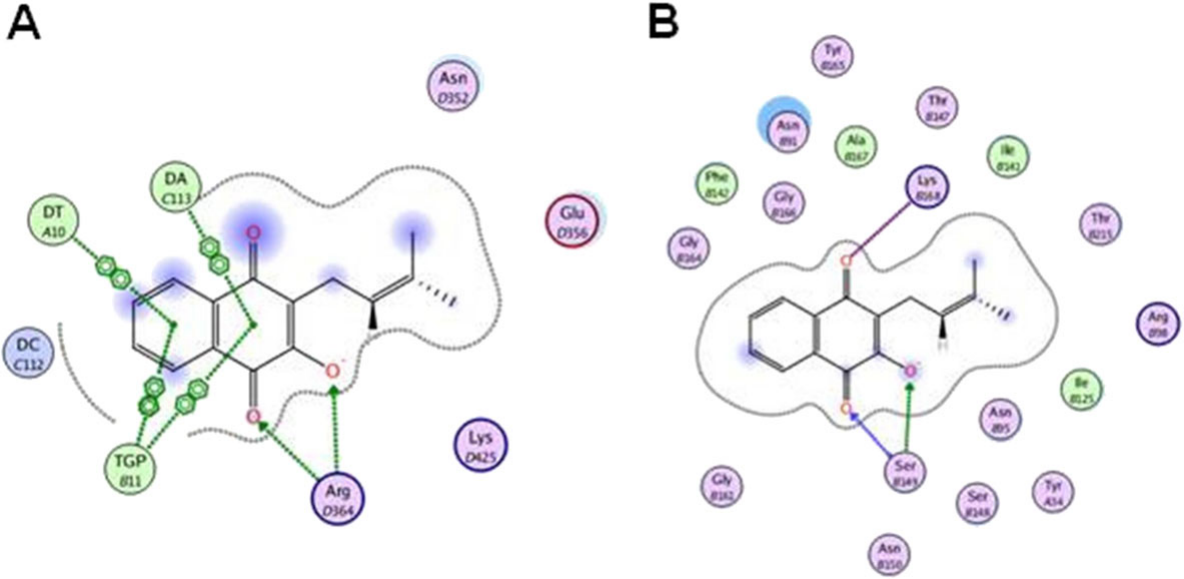
The molecular docking for the interaction modes of lapachol-TOP I (**a**) and lapachol-TOP II (**b**). MOE 2010 software package was used for docking TOP I and II. Crystal structures of TOP I-ligand complex (PDB entry: 1SC7, 3.0 Å) and TOP II-ligand complex (PDB entry: 1ZXM, 1.87 Å) were used. The scores of lapachol-TOP I and lapachol-TOP II were -12.59 and -6.71, respectively

Molecular docking study was performed to better understand the possible interaction mode of lapachol-TOP I and lapachol- TOP II. The structures of Topo I and II used in the docking study were obtained from the Protein Data Bank (PDB entry: 1SC7 and 1ZXM). To verify whether MOE software was suitable for docking TOP I and TOP II, the RMSD values of docking conformation and initial conformation were calculated. In general, if RMSD were less than 2.0 Å, MOE software was recognized as suitable for docking study. RMSD of TOPO I and II calculated in the present study were 1.022 and 0.779 Å, indicating that MOE software can better simulate the action mode of ligands and TOP I or TOP II in the crystal structures. The scores of initial ligands and the protein crystal structures were -17.78 and -11.43, respectively. The scores of lapachol-TOP I and lapachol-TOP II were -12.59 and -6.71, respectively. The molecular docking modes were shown in Fig. 7. Lapachol and TOP I showed relatively stronger interaction than that of the initial ligand-protein crystal structures, with more conjugate and hydrogen bond actions. Lapachol and TOP II showed relatively weak interaction, with only hydrogen bond actions.

#### Lapachol could inhibit the expressions of TOP II in C6 cells

**Fig. 8:**
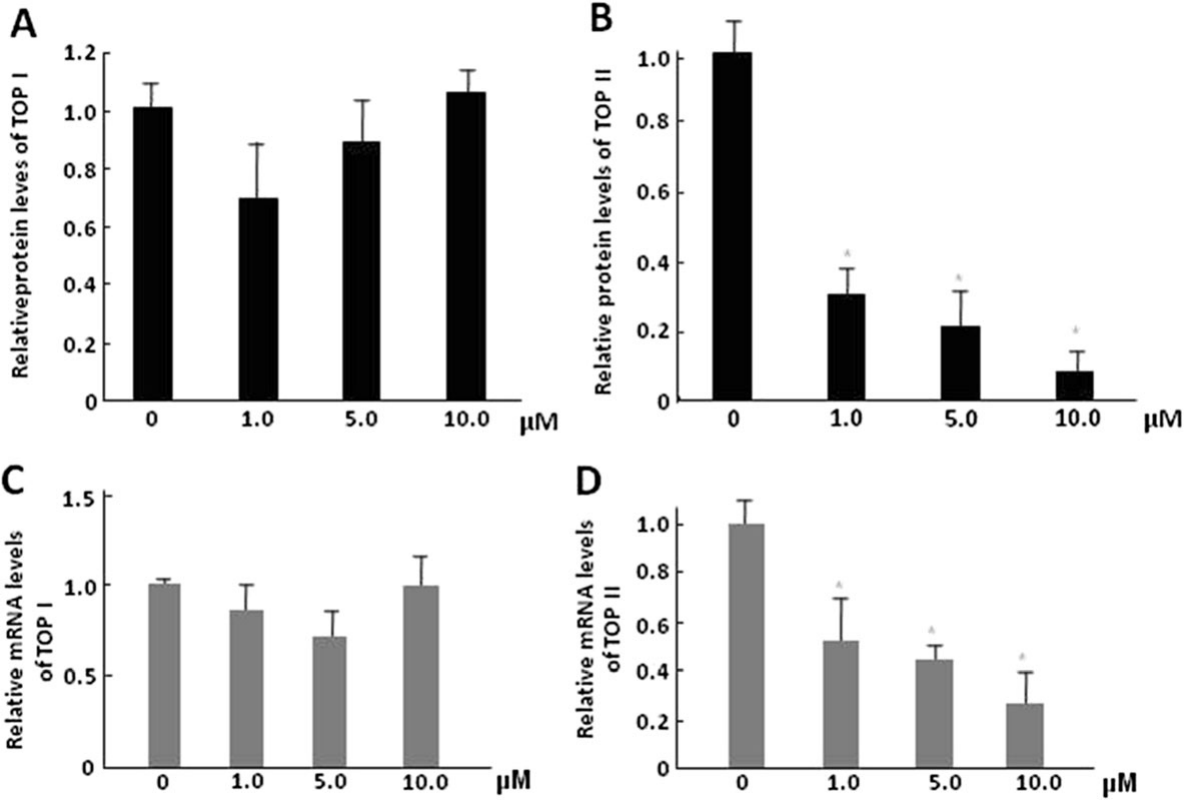
The relative contents of TOP I and TOP II in C6 glioma cells after being treated with control, 1 μM lapachol, 5 μM lapachol, and 10 μM lapachol for 48 h. **a** relative TOP I protein level by ELISA; **b** relative TOP II protein level by ELISA; **c** relative TOP I mRNA level by RT-PCR; **d** relative TOP II mRNA level by RT-PCR. Mean ± SD of three independent experiments. *P < 0.05 compared with control

TOP I and TOP II expression levels in the cells were determined by Enzyme-linked Immunosorbent Assay Kit. The result showed that lapachol could significantly inhibit TOP II expression in C6 cells in a dose dependent manner (*P* < 0.05), but not TOP I expression. After treatment with 1.0, 5.0, and 10.0 μM lapachol, the TOP II expression in C6 cells decreased to 0.31, 0.22 and 0.09 times of that of the control (Fig. 8b). However, lapachol showed no obvious inhibitory effects on TOP I expression levels in C6 cells (Fig. 8a). RT-PCR was performed for detecting the mRNA expression levels of TOP I and TOP II. The results showed that lapachol could significantly inhibit mRNA levels of TOP II in a dose dependent manner (*P* < 0.05), but not TOP I (*P* > 0.05) (Fig. 8c and d). After treatment with 1.0, 5.0, and 10.0 μM lapachol, the TOP II mRNA levels in C6 cells decreased to 0.61, 0.44 and 0.23 times of that of the control (Fig. 8d).

## Discussion

Lapachol is a naturally occurring naphthoquinone derived from Bignoniaceae (Tabebuia sp.) that possesses various activities, including anti-inflammatory, antibiotic, antifungal, antitumor and immunomodulatory [19]. Antitumor effects of lapachol have been extensively studied for many years. The cytotoxicity of lapachol and its derivatives were evaluated in many tumor cells and several mouse models [8–11]. It was reported that lapachol showed strong cytotoxicity on glioma cells [10]. We have previously found that lapachol was able to cross the blood brain barrier, indicating that it might be effective in treating malignant glioma [17]. Thus, in this study we evaluated the inhibitory effects of lapachol on malignant glioma both in vitro and in vivo. MTS/PMS assay showed that lapachol exhibited strong inhibitory effects on C6 cells in a dose dependent manner, with the IC_50_ of 3.7 ± 1.4 μM. The rat C6 glioma model was established for the in vivo experiment. The results showed that tumor volumes in the brains of the rats in the high dose lapachol and TMZ treated groups were significantly decreased compared with the control group (*P* < 0.05) (Fig. 3a). However, no obvious growth inhibitory effects were observed in the low dose lapachol and middle dose lapachol groups compared with the control group (*P* > 0.05). Moreover, no obvious body weight changes were found in lapachol and TMZ treated groups compared to the control group (*P* > 0.05) (Fig. 3b). The result suggested that high dose lapachol and TMZ treatment could significantly decrease C6 glioma growth without affecting the body weights of the glioma-bearing rats.

Most current chemotherapeutic agents act through the activation of the apoptosis signal pathway [20]. Our hoechst 33258 staining and Annexin V-FITC/PI staining results indicated that lapachol could significantly induce the apoptosis of C6 cells in dose dependent manners (Fig. 5a  and b). We also measured DNA damage caused by lapachol using comet assay. The results indicated that lapachol could induce DNA damage in a concentration-related manner (Fig. 5c and d). It was reported that p53 mutations was closely related with the high proliferation rate of glioblastoma [21]. Asai A, et al. investigated the expression of p53 in several human (U251, U87, U343) and rat glioma cell lines (C6, 9 L) and found that U87, U343, and C6 cells expressed wild type-p53 messages while U251 and 9 L cells expressed mutated form-p53 messages [22]. So, wild-type p53 may increase and mediate the multiple cellular responses for DNA damage repair or apoptosis induced by lapachol. Further studies are needed to confirm this hypothesis.

DNA topoisomerases have been identified as targets for drug development and some TOP I or TOP II inhibitors have already been used in clinic [23]. Many studies showed that the antitumor activity of naphthoquinone was associated with inhibition of DNA topoisomerase activities [13, 14]. Since lapachol belongs to naphthoquinone, we first detected the effects of lapachol on TOP I and TOP II activities by the conversion of supercoiled pBR322 DNA to its relaxed form. The results showed that lapachol exhibited obvious TOP I and TOP II inhibitory activities (Fig 6). Also the result showed that lapachol act as TOP II poisons (Fig. 6b). The effect of lapachol on TOP II activity is consistent with previous studies [9]. However, the effect of lapachol on TOP I activity is firstly reported.

To better understand the possible interaction modes of lapachol-TOP I and lapachol-TOP II, molecular docking study was performed. The result showed that lapachol-TOP I showed relatively stronger interaction, with more conjugate and hydrogen bond actions, while lapachol-TOP II showed relatively weak interaction, with only hydrogen bond actions (Fig. 7). The reason why lapachol act as a TOP II inhibitor may be caused by the interaction between lapachol and DNA molecular. We also evaluated the effects of lapachol on TOP I and TOP II expression levels in the cells by Enzyme-linked Immunosorbent Assay and RT-PCR. The results showed that lapachol could significantly inhibit the protein and mRNA levels of TOP II in a dose dependent manner, but not TOP I levels (Fig. 8). Most TOP I and TOP II inhibitors could inhibit the activities of TOP I and TOP II, but not the expression levels [13, 14]. Further studies are needed to compare the inhibitory levels of lapachol on TOP I and TOP II with different concentrations of other TOP I and TOP II inhibitors.

TOP II has become an attractive target for cancer therapies and TOP II inhibitors are among the most effective anticancer drugs [24]. Most of the currently used TOP II-targeted drugs, such as mitoxantrone and doxorubicin, cause DNA damage and cell apoptosis [24]. Almost all TOP II inhibitors in clinical use are TOP II poisons, including etoposide, doxorubicin, and mitoxantrone. In this study we also found that lapachol acted as TOP II poisons.

## Conclusion

These results showed that lapachol could significantly inhibit C6 glioma both in vitro and in vivo, which might be related with inhibiting TOP I and TOP II activities, as well as TOP II expression. The current study suggested that lapachol should be further explored for the potential use in malignant glioma therapy.
